# Mechanisms of sterile inflammation after intravitreal injection of antiangiogenic drugs: a narrative review

**DOI:** 10.1186/s40942-021-00307-7

**Published:** 2021-05-07

**Authors:** William J. Anderson, Natasha Ferreira Santos da Cruz, Luiz Henrique Lima, Geoffrey G. Emerson, Eduardo Büchele Rodrigues, Gustavo Barreto Melo

**Affiliations:** 1 Department of Ophthalmology, Saint Louis University School of Medicine, Saint Louis, MO USA; 2 Department of Ophthalmology, Federal University of São Paulo, São Paulo, Brazil; 3 Retina Center of Minnesota, Minneapolis, MN USA; 4 Hospital de Olhos de Sergipe, Rua Campo Do Brito, 995, Aracaju, SE 49020-380 Brazil

**Keywords:** Abicipar, Aflibercept, Bevacizumab, Faricimab, Intravitreal injection, Noninfectious inflammation, Pegaptanib, Ranibizumab, Silicone oil, Sterile endophthalmitis

## Abstract

**Background:**

Intraocular inflammation is an uncommon but potentially vision-threatening adverse event related to anti-VEGF therapy. This is of increasing importance given both the volume of injections performed, as well as the increased prevalence of inflammation seen with newer anti-VEGF agents. Brolucizumab, the newest anti-VEGF agent, has been associated with an inflammatory retinal vasculitis and the underlying mechanism is unclear. Reviewing potential mechanisms and clinical differences of intraocular inflammation may assist clinicians and scientists in reducing the risk of these events in the future.

**Observations:**

Two types of inflammation are seen with intravitreal injections, acute onset sterile inflammation and delayed onset inflammatory vasculitis. Acute onset inflammation can be subcategorized into subclinical anterior chamber inflammation and sterile uveitis/endophthalmitis. Subclinical anterior chamber inflammation can occur at rates as high as 19% after intravitreal anti-VEGF injection. Rates of sterile uveitis/endophthalmitis range from 0.05% to 4.4% depending on the anti-VEGF agent. Inflammatory vasculitis is only associated with brolucizumab and occurred in 3.3% of injections according to the post hoc review of the HAWK/HARRIER data. In addition, silicone oil from syringes can induce immunogenic protein aggregates. Agitation of the syringe, freeze thawing, shipping and improper storage prior to injection may increase the amount of silicone oil released from the syringe.

**Conclusion:**

The main factors which play a role in intraocular inflammation after anti-VEGF injection can be divided into three causes: patient-specific, medication-specific and delivery-specific. The majority of clinically significant inflammation seen after intravitreal injection is an acute onset inflammatory response with most patients recovering baseline VA in 3–5 weeks. The presence of pain, hypopyon, severe anterior chamber reaction, hyperemia and significant vision loss may help distinguish infectious from non-infectious etiologies of post injection inflammation. Avoiding temperature fluctuation, mechanical shock, agitation during transport and handling of syringes/drugs, and the use of SO-free syringes may help minimize intraocular inflammation. While a definitive mechanism has not yet been established, current knowledge of the clinical presentation and vitreous histopathology of brolucizumab-retinal vasculitis favors an auto-immune type IV hypersensitivity reaction.

## Introduction

Intravitreal injections of anti-vascular endothelial growth factor (anti-VEGF) are the most commonly performed intraocular treatment worldwide [1]. The number of indications for their use has increased throughout the years, as anti-VEGF therapy has become the mainstay of treatment for several common retinal diseases including neovascular age-related macular degeneration (AMD). Rarely, this treatment may lead to adverse events such as intraocular hemorrhage, retinal detachment, endophthalmitis and intraocular inflammation. Of these complications intraocular inflammation (IOI) is of particular interest as increased rates have been noted with more recent antiangiogenic drugs. This inflammation may range from a mild transient reaction to potentially vision-threatening outcome after intravitreal injection of anti-VEGF therapy [2]. Since it may be easily mistaken for infectious endophthalmitis, a clear understanding of this pathology is essential. Infectious endophthalmitis is usually associated with pain, hypopyon, severe anterior chamber reaction, hyperemia and significant vision loss, while symptoms associated with sterile endophthalmitis are less severe [3]. In order to decrease the rates of inflammation, it is critical to consider all factors that may play a role both in clinical practice and pharmaceutical development.

The current intravitreal VEGF inhibitors in clinical use are bevacizumab (Avastin, Genentech, San Francisco, CA), ranibizumab (Lucentis, Genentech, San Francisco, CA), aflibercept (Regeneron, Tarrytown, NY), and brolucizumab (Beovu, Novartis, Basel, Switzerland) [4–8]. Bevacizumab was first approved for use in the treatment of colorectal cancer, however it is used off label for intraocular use. It is repackaged by compounding pharmacies for intravitreal injection, and has been shown to have similar efficacy and safety profile when compared to ranibizumab [9]. Of note, pegaptanib (Macugen, Eyetech/OIS Pharmaceuticals, Melville, NY) was in fact the first anti-VEGF therapy approved for intravitreal injection in 2004, however it is no longer utilized due to the superior efficacy of newer anti-VEGF agents [10]. Each anti-VEGF agent may pose a higher or lower risk of inflammation based on different mechanisms associated with each drug. The overall incidence of sterile intraocular inflammation mimicking uveitis/endophthalmitis post injection has varied widely in the literature, with ranges from 0.005%–4.4% [11–13].

We aim to review previous research regarding intraocular inflammation associated with anti-VEGF therapy while discussing recent developments into the role of silicone oil (SO) in the inflammatory process. We also discuss inflammatory vasculitis observed with recently released brolucizumab, including potential mechanisms. Collectively our review will provide further insight into the mechanisms and clinical differences among various types of inflammation, with the goal that clinicians and scientists can reduce the risk of these events in the future. This topic is of importance given the increased rates of inflammation seen with brolucizumab, as well as the recent FDA rejection of abicipar pegol (Abicipar, Allergan, Dublin, Ireland) due to high rates of intraocular inflammation.

A literature review was performed using PubMed to identify relevant English-language articles published through March 25, 2021. Search terms included anti-VEGF, inflammation, sterile endophthalmitis, uveitis, intravitreal injections, sterile inflammation. Papers unrelated to the topic of this review papers were excluded. The final literatures resulted in 84 total articles. Due to the lack of randomized clinical trials, the authors also included case reports, case series, and review articles. The authors reviewed the titles and abstracts for inclusion. Additional relevant articles were identified from the review of citations referenced as well as from the similar papers of the Pubmed website search.

## Clinical features and epidemiology of inflammation after antiangiogenic injection

Inflammation associated with intravitreal anti-VEGF injection manifests with a broad range of clinical features which may be generally categorized into two mechanisms. The first mechanism results in an acute onset sterile inflammation. This type of inflammation occurs on a spectrum, ranging from subclinical anterior chamber inflammation to significant inflammation mimicking endophthalmitis. The second mechanism is a delayed onset inflammatory vasculitis which has been described with brolucizumab. The overall inflammatory rates vary between specific anti-VEGF agents and a variety of outside factors may influence observed rates of inflammation.

### Acute onset sterile inflammation

The timing of acute onset sterile inflammation following intravitreal anti-VEGF agents is rapid, typically within the first 5 days of injection [2, 11, 14]. It may present as a transient subclinical anterior chamber reaction, usually occurring in the first days immediately following IVI. Cell and flare can typically be observed in the anterior chamber on slit lamp biomicroscopy. If the patient is being seen at monthly intervals, this type of inflammation may often go unnoticed as the patient is usually asymptomatic. No intervention is necessary for this transient, asymptomatic inflammation. The highest rates of subclinical anterior chamber inflammation have been seen with aflibercept (19%), compared to much lower rates seen with ranibizumab (2%) [15].

Acute sterile inflammation can occasionally be more severe, presenting as a sterile uveitis/endophthalmitis. The variable clinical presentation of post-injection sterile uveitis/endophthalmitis most commonly includes decreased visual acuity (93%), vitreous cells (81%), anterior chamber cells (74%), and floaters (60%). Less commonly, corneal edema, conjunctival injection, ocular pain, photophobia, hypopyon, keratic precipitates, trabeculitis, retinal infiltrates and hemorrhages may occur [2, 11, 12, 14, 16–19]. Overlapping characteristics make distinguishing sterile uveitis/endophthalmitis from infectious endophthalmitis difficult at times, however differences in presentation are highlighted in Table 1 [20, 21]. Severe pain, hypopyon, and hyperemia are more common with infectious endophthalmitis.

In sterile uveitis/endophthalmitis the mean visual acuity usually drops at presentation but often recovers after 1 month [2, 11, 19]. It is important to note that recurrent post-injection inflammation was found to be uncommon in previous studies [2, 11, 14, 22]. Cases of sterile endophthalmitis may be observed or treated with topical corticosteroid drops. Systemic or periocular steroids are rarely utilized [2, 11, 14, 23, 24].

The frequency of sterile uveitis/endophthalmitis inflammation after intravitreal injection is relatively rare, and documented incidence rates with each anti-VEGF therapy are variable (Table 2) [25]. Pegaptanib, the first anti-VEGF therapy utilized, had 16% of patients experience a mild anterior chamber reaction after injection with 0.3 mg dosing in the VISION trial, higher than the rate of 6% seen in the sham injection group [26]. Sterile endophthalmitis was not reported in the VISION trials of pegaptanib. Bevacizumab has been shown to have an incidence of sterile inflammation between 0.09% and 1.1% according to several retrospective studies [16, 27, 28]. Inflammatory reactions have also been reported after intravitreal injection of ranibizumab, however the reported rates of inflammation have typically been lower [29]. The MARINA and ANCHOR trials with ranibizumab showed an incidence of 0.05% [30, 31], and a subsequent comparison study of ranibizumab and bevacizumab for neovascular AMD reported rates of 0.16% for ranibizumab and 0.5% for bevacizumab [32].

The phase III VIEW trials of aflibercept did not specifically categorize inflammation or uveitis as an adverse event, however rates of vitreous floaters and endophthalmitis were not statistically different from ranibizumab [33]. Within the first 3 months of approval, a cluster of cases of intraocular inflammation occurred with aflibercept, with 15 cases reported during that time lapse. The American Society of Retina Specialists (ASRS) released a statement with detailed information surrounding these events, and no identifiable cause was found [34]. A large retrospective review reported rates of sterile endophthalmitis with aflibercept to be 0.16%, compared to 0.10% for bevacizumab and 0.02% with ranibizumab [12].

Newly FDA-approved brolucizumab has shown higher rates of clinically significant inflammation when compared to aflibercept in the HAWK/HARRIER clinical trials (4.4% versus 0.3% with aflibercept) [7]. The majority of these cases of inflammation were a mild, transient sterile iritis/uveitis similar to inflammation seen with previous agents.

At the time of this review, the FDA has declined the approval of a novel anti-VEGF agent abicipar pegol (Abicipar; Allergan, Troy Hills, NJ, USA) for the treatment of neovascular AMD due to a high rate of IOI observed with the drug. Inflammation in the initial CEDAR/SEQUOIA trials were 15.4% and 15.3% [35]. This was mostly a mild to moderate uveitis/iritis, however 1.8% of patients developed retinal vasculitis [35]. The total rate of IOI was 8.9% in the MAPLE trial after reformulation [36].

### Delayed onset inflammatory vasculitis

The second type of inflammation seen with intravitreal anti-VEGF is a delayed onset retinal vasculitis. The only FDA approved anti-VEGF agent which has currently been associated with inflammatory vasculitis is brolucizumab. Several reports of retinal vasculitis and/or retinal vascular occlusion were reported after the FDA approval of brolucizumab, and in response Novartis commissioned an independent Safety Review Committee (SRC) to review all of the data in the phase 3 HAWK/HARRIER trials. In this post hoc review it was found that this inflammation and/or vasculitis can occur as far out as 12–18 months, although the majority (~ 75%) occurred within 0–6 months(13) (13) (13) (13) (13) (13) (13) (13) (13) (13) (13) (13) (13) (13) (12) (12) (11). The rate of inflammation and concomitant retinal vasculitis was reported at 3.3%, while the rate of vascular occlusion was 2.1%. Of patients who developed retinal vasculitis, approximately 22% experienced at least moderate vision loss (> 15 ETDRS letters lost). The total incidence of moderate vision loss due to IOI was less than 1% with brolucizumab, and the overall rate of moderate vision loss in the study was similar between brolucizumab and aflibercept (13) (13) (13) (13) (13) (13) (13) (13) (13) (13) (13) (13) (13) (13) (12) (12) (11).

The largest study outside of the clinical trials to date evaluating retinal vasculitis after brolucizumab is a retrospective analysis of 26 eyes from 25 patients that were reported to the ASRS [37]. This study showed that BCVA worsened from an average baseline of 20/52 to 20/151 at time of presentation. Average time to presentation was 53 days after the last brolucizumab injection (range 8–137 days). At final follow up, mean final BCVA was 20/243 and 46% had a greater than 3-line decrease in visual acuity. Clinical presentation was variable, however 92% of eyes exhibited signs of either anterior inflammation and/or posterior inflammation. Anterior chamber cell was present in 65% of eyes. Patients had variable extent of retinal vasculitis, with involvement of retinal arteries, veins or choroidal vessels. Choroidal ischemia was noted in 10 of 21 eyes. Occlusive vasculitis was noted in 85% of eyes [37].

A standardized treatment has yet to be established for this vasculitis secondary to intravitreal brolucizumab, but cases reported in the literature to date have been managed with a variety of steroid therapies (topical, oral, sub-tenon, intravitreal) or observation [38–40]. Vitrectomy was performed in 2 cases with no improvement in vision [38]. Some patients had improvement in visual acuity with steroid treatment, however cases where significant retinal artery occlusion occurred had little to no improvement with steroid therapy.

In summary, there are two types of sterile inflammation seen in association with intravitreal anti-VEGF therapy. Table 2 highlights the characteristics of both acute onset sterile inflammation and delayed onset inflammatory vasculitis associated with intravitreal anti-VEGF injections. Acute onset inflammation can range from subclinical anterior chamber inflammation to sterile uveitis/endophthalmitis. Subclinical anterior chamber inflammation may occur at rates as high as 20% after intravitreal anti-VEGF injection. Rates of sterile uveitis/endophthalmitis range from 0.05% to 4.4% depending on the anti-VEGF agent. Inflammatory vasculitis is only associated with brolucizumab and occurred in 3.3% of patients in the HAWK/HARRIER trials according to post hoc analysis [13].

## Putative causative factors of acute onset sterile inflammation

**Fig. 1:**
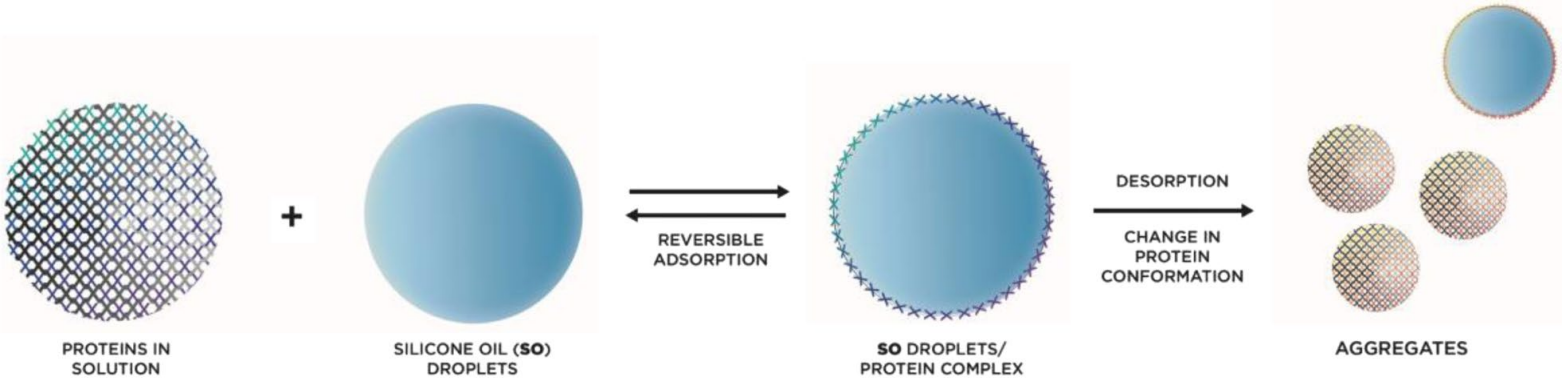
Schematic drawing illustrating possible interactions between silicone oil (SO) and proteins in solution. Proteins may undergo conformational change and film formation after interaction with the silicone oil surface. Fragmentation of SO-protein complexes results in smaller aggregates and agglomerates

There are a number of known and suspected causes that may contribute to inflammation following intravitreal injections. While the exact mechanism for sterile uveitis/endophthalmitis may not be clear for each individual case, knowledge of potential causative factors can allow us to pursue interventions aimed to decrease this occurrence. Numerous investigations and studies have identified several factors that may play a role in this phenomenon (Fig. 1).

### Patient susceptibility

The idea that individual patients may be more prone to an immunologic reaction to intravitreal injections is mostly conjectural, however it has been shown that a small subset of patients have anti-idiotype antibodies against anti-VEGF antibody [27]. The presence of anti-drug antibodies (ADA) may also occur with any anti-VEGF agent. Treatment-naïve patients may exhibit baseline ADA, but more commonly transient ADA occurs after administration of the drug. The presence of ADA is available for each drug during clinical trials.

No evidence of baseline serum anti-pegaptanib IgG and IgM antibodies was detectable throughout the first year of treatment in the VISION trial [10]. Ranibizumab anti-Fab immunoreactivity in treatment-naïve patients was between 0–0.9% in the MARINA and ANCHOR trials [30, 31]. Baseline aflibercept ADA were detected at rates between 1–3% in the VIEW trials [33]. Brolucizumab exhibited high rates (43.7%) of ADA in treatment-naïve patients [41]. However, this appears to be consistent with rates of ADA seen in antibody fragments of similar structure such as nanobodies and single domain antibodies. This may be a factor as to why increased IOI was observed with brolucizumab, as the EMA did find a correlation found to incidence of intraocular inflammation and elevated titers of ADA in clinical trials with brolucizumab [38, 41, 42]. There were low levels of treatment-naïve ADA with abicipar when evaluated by the EMA (0.7%), yet very high levels of observed intraocular inflammation [43]. Other factors may be at play with abicipar such as impurities in the manufacturing process, *E. coli* remnants in the drug product, post-translational protein modification, or other causes. Regardless of anti-VEGF agent, pre-treatment ADA titers have not been shown to have any clinically relevant difference in BCVA outcomes [30, 33, 41, 43].

In addition, patients may theoretically be more prone to inflammation when breakdown of the blood-retina barrier occurs in certain conditions such as exudative AMD, compromising the immune privilege of the vitreous [44]. Other presumptive factors that may predispose patients to develop inflammation include the use of pro-inflammatory drugs, such as prostaglandins, or a known history of inflammation from uveitis [2].

### Anti-VEGF medication

Several factors of the anti-VEGF medication or its associated suspension are postulated to induce an inflammatory response. Anti-VEGF agents are manufactured using biologic recombination using recombinant DNA technology either in *E. coli* bacteria or Chinese hamster ovary (CHO) cell lines [45, 46]. Noninfectious contamination during this process, including endotoxins, can cause serious inflammatory complications. This was well documented in a previous outbreak of endotoxin mediated inflammation related to counterfeit bevacizumab [47]. While endotoxin levels were abnormally high in the counterfeit drug, there still remains the possibility that very low levels of endotoxin or immunogenic proteins could contribute to an inflammatory response. This theory has been corroborated in animal studies [48].

Inflammation specifically related to bevacizumab may be partially due to its formulation for intravascular use rather than intravitreal use. Wickremasinghe et al. described how bevacizumab preparations originally designed for intravascular use may contain traces of endotoxin that result in intravitreal inflammation even though it may cause no inflammation when administered intravascularly [27].

The manufacturing process could also be a significant contributor and may explain higher inflammatory rates seen in newer anti-VEGF therapies such as abicipar pegol. Rates of inflammation with abicipar pegol dropped from roughly 15% in the CEDAR and SEQUOIA clinical trials to 8.9% in the MAPLE clinical trial after reformulation [49, 50]. The complex biologic processes that occur in the manufacturing of these antibodies may allow for impurities or non-human proteins to play a role in inflammation caused by these agents. The immunogenicity of these newer agents may continue to improve as drug companies continue to purify the extraction process of these medications.

In addition, the formulation in which the drug is administered has previously been shown to induce an inflammatory response. Initial studies on ranibizumab showed an 11.4% inflammation rate, which drastically improved after reformulating the drug from a lyophilized formulation to a solubilized formulation [28, 51].

Lastly, the actual anti-VEGF antibody itself may have immunogenic properties. It has been shown that higher rates of inflammation occur with aflibercept and bevacizumab compared to ranibizumab [12]. One potential explanation for this occurrence is an inflammatory reaction triggered by the Fc antibody portion present on aflibercept and bevacizumab, which is absent on the ranibizumab molecule. This proinflammatory Fc portion can interact with intraretinal Fc receptors and may be a contributing factor to higher documented rates of inflammation. This theory has been supported by increased expression of intraretinal Fc receptors from donor eyes with neovascular AMD [52]. This knowledge has been applied in the development of faricimab (Genentech, San Francisco, CA), which is currently under investigation for its use in wet-AMD and diabetic macular edema. Faricimab is a novel bispecific antibody currently in Phase III trials which independently binds VEGF-A and angiopoietin 2 via two separate antibody fragments bound by a modified Fc region. The Fc portion of this antibody has been optimized to eliminate binding of neonatal Fc and Fcγ receptors, decreasing both inflammation and systemic exposure. The results thus far have been promising, while Phase III trials have yet to be published, inflammation associated with faricimab in Phase II trials have been comparable to rates seen with ranibizumab [53, 54].

### Silicone oil and protein aggregation

Protein aggregation or change in conformation has been studied more recently as a possible etiology of sterile uveitis/endophthalmitis [55, 56]. Protein aggregates can form under a variety of circumstances prior to intravitreal injection of the drug and they can be further induced by SO release from syringes [57–59]. The inner cylinder wall of most syringes commonly used for intravitreal injection are siliconized to decrease plunger resistance. Various factors can contribute to SO release from the inner cylinder wall, protein denaturing, or both [55, 57–59]. It is possible that SO-protein complexes injected into the vitreous could have the potential to invoke an immunogenic response, as it has been demonstrated in lab studies in animals and cells [60, 61].

Many processes involved in the shipping, handling and storage of anti-VEGF medications and syringes can contribute to SO release and protein aggregation [55, 57–59]. Temperature is one variable which can cause this event. Freeze-thawing that may occur during shipping has been demonstrated to significantly increase SO microdroplet release in bevacizumab solution, along with increased protein particle levels [58]. In contrast, high temperatures may also promote particle formation, protein unfolding and aggregation [62, 63]. Exposure to light has been shown to increase particulate count by 2.5 times [58].

Agitation of the syringe itself can also induce SO release from syringes [64–66]. This may occur during shipping but most frequently occurs during preparation, as physicians flick the syringe prior to injection to separate air from the solution [58, 64–66]. Our authors have recently shown that this agitation by flicking or tapping usually used to separate air from the medication leads to significant release of SO by syringes [64]. The amount of SO released from syringes varies based on syringe manufacturer [65]. We have also published a case–control study that associated inflammation after intravitreal injection of aflibercept and the use of a specific syringe brand, Saldanha-Rodrigues (SR) syringes [56]. In this series of patients, inflammation occurred in all patients who received intravitreal injection after agitation of the SR syringe by flicking prior to injection. The control group that received aflibercept injection with the SR syringe and no agitation did not develop inflammation [56]. Other studies unrelated to ophthalmology also corroborate the hypothesis that agitation provides greater release of oil from the inner wall of the syringe [67, 68].

**Fig. 2:**
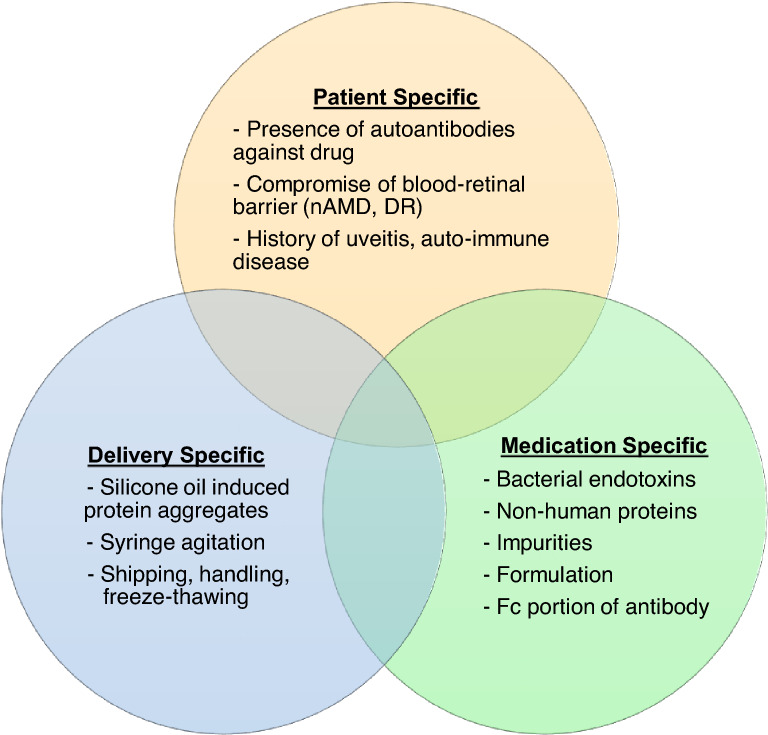
Contributing factors of inflammation

All of these processes that result in SO-protein complex formation as well as protein aggregates can, in turn, elicit an immune response [61, 69]. Proteins in solution may interact with silicone oil in a way that results in post-translational protein modification, altering the proteins and creating aggregates (Fig. 2). Even small non-visible SO droplets have been shown to have the potential to create these potentially immunogenic protein complexes. Previous studies have shown that micron and submicron sized SO droplets have the ability to form SO-protein complexes with monoclonal antibodies and stimulate an immunogenic reaction [60, 61]. Imaging techniques utilized by Probst also confirmed the ability of the micron and submicron sized SO droplets to induce protein complexes and aggregates [70]. These studies suggest that even small amounts of silicone oil may interact with the anti-VEGF molecules, and this may result in potentially immunogenic alterations of these antibodies. While silicone oil induced protein aggregation may theoretically affect all proteins, studies have shown variable aggregation based on protein structure. Further investigation is necessary as to whether this phenomenon preferentially effects certain anti-VEGF agents more than others.

## Immunology and possible pathways of silicone oil/protein involvement in inflammation

The origin and identification of anti-VEGF agents as self or external by the immune system are important factors that influence their immunogenicity [71, 72]. Another known factor is the immunological tolerance to self-proteins. Pronounced immune response may follow inoculation of aggregated types of external therapeutic proteins, while aggregated compositions of therapeutic endogenous proteins can result in weak immune reaction. [72] Extrinsic epitopes on the anti-VEGF molecule may be presented by antigen-APCs to B- and T-cells, triggering antibody production and generation of memory cells that develop an enhanced immune reaction [73]. In addition, physical (protein misfolding or aggregation caused by improper production, dosage or storage status) and chemical (oxidation) protein deterioration may be involved in the generation of a more immunogenic protein [73].

SO microdroplets can behave as immunological catalysts, causing an antibody response against a recombinant self-protein, and the capability of these microdroplets to perform as adjuvants and amplify antibody reactions against injected therapeutic proteins may be related to these proteins inherent immunogenicity [69]. Chisholm CF et al. pointed out that formulations of recombinant murine growth hormone (rmGH) that included SO microdroplets when administered daily to mice may decrease the immunological tolerance to a recombinant self-protein, and the frequency of therapeutic drug injection is also related to immunological tolerance breakage to a recombinant self-protein. The inherent protein immunogenicity may determine the adjuvant potency of SO, with foreign proteins inducing more robust effects [74]. In another report, using injections of hen egg lysozyme (HEL) formulated in the presence and absence of SO microdroplets in both wild-type mice and transgenic littermates, Chisholm CF et al. showed that adsorption of proteins to SO could change the protein structure [75]. Furthermore, the presence of SO microdroplets performed as an adjuvant to enhance the immune reaction against HEL. This ancillary effect did not develop in the setting of protein and SO injected at different sites, conjecturing that the adsorption of protein to SO or the co-localization of both should be critical for the expression of SO adjuvant effect [75].

An intraocular injection of a therapeutic agent associated with aggregated protein is a major concern as protein aggregates are able to induce an immune response [76]. The switch of an antigenic stimulation into an immunologic response develops by provoking naive B and T lymphocytes and comprises an interplay adjustment between antigen and naive lymphocytes. The result of this process is the lymphocyte sensitization, and, subsequently, immunoglobulin G (IgG) antibodies are released by B lymphocytes. IgG has a significant role in immune response, and the expression of a specific IgG is correlated to antibody response maturation. IgG can attach to various types of microorganisms, such as virus, fungus and bacteria, or foreign proteins, defending the eye against them by means of complement activation, phagocytosis, agglutination and neutralization of their toxins. The altered immune response to protein formulations that have SO microdroplets is probably associated with a T-cell-dependent B cell activation process related to IgG1 production [75, 77, 78]. Therefore, the T-cell-dependent immune reaction against a foreign protein antigen could explain the IgG expression with consecutive development of intraocular inflammation following intravitreal injection of therapeutic proteins in conjunction with silicone oil.

Although the precise mechanism of acute onset sterile inflammation after intravitreal injection of antiangiogenic drugs is likely multifactorial, a new theory suggests that protein aggregation due to syringe agitation or interaction between protein molecules and SO may contribute to this inflammatory reaction.

## Immunology and pathways of late onset inflammatory vasculitis

In addition to acute sterile inflammation, brolucizumab has been associated with a delayed onset retinal vasculitis. The exact etiology for this inflammatory response has not been elucidated but is most likely related to a delayed hypersensitivity reaction [38, 39]. The female predominance of brolucizumab-associated retinal vasculitis reported by Witkin et al. (22 of 25 patients were female) may point towards an auto-immune response, although no definitive correlation to auto-immune disease has been made [37]. The small number and heterogeneity of cases to date has made clarifying a causative factor difficult. Several reports have postulated either a type III or type IV hypersensitivity reaction [38, 79]. Type III hypersensitivity reactions have been known to occur due to intravascularly injected monoclonal antibodies [80]. Additionally, there are several similarities between brolucizumab associated retinal vasculitis and vancomycin associated hemorrhagic occlusive retinal vasculitis (HORV) [38, 81].

HORV was previously thought to have been secondary to a type III hypersensitivity reaction [81]. However, more recent literature of the histopathology related to the disease has indicated this is may be a T-cell predominant type IV hypersensitivity [82]. Similar to the described reaction with brolucizumab, HORV has also been found to have choroidal involvement, and it has even been proposed that hemorrhagic occlusive choroidal retinal vasculopathy (HOCRV) would be a more appropriate terminology [82]. The involvement of the choroid in both HORV and brolucizumab-associated vasculitis would not be expected in either a sterile uveitis or a pure vasculitis. The lack of retinal hemorrhages seen in the vasculitis related to brolucizumab versus HOCRV may be related to arterial occlusion and/or VEGF inhibition. Hemorrhages may develop later in the clinical course, likely as the effects of VEGF inhibition diminish [38]. A recent case report analyzing a vitreous sample obtained during vitrectomy of a patient with brolucizumab-related retinal vasculitis showed the presence of CD3, CD4, CD8, and CD68. These findings indicate the presence of T cells and histocytes, favoring a type IV reaction [42]. However, the presence of both B and T cells may indicate a mixed type III and IV reaction [42].

The timing of onset also points towards a delayed hypersensitivity reaction, where repeat exposure may result in a more rapid immune response. The average time to presentation in recent case series has ranged from 30 to 53 days after injection [37, 38]. However, Baumal et al. reported that retinal vasculitis occurred earlier in patients who had received more than one IVI of brolucizumab compared those who had a reaction to the first injection (20 days post IVI vs 35.5 days post IVI, respectively) [38]. The exact immunologic trigger for this reaction to brolucizumab and not to other anti-VEGF agents still remains unknown but may be due to a variety of factors.

Brolucizumab and abicipar-pegol, the only two anti-VEGF therapies associated with retinal vasculitis, are characteristically smaller molecules than the rest of the anti-VEGF therapies currently in use. Brolucizumab is unique in the fact that the small size of the molecule allows for higher molar concentration, better tissue penetration, and a prolonged therapeutic effect compared to other anti-VEGF agents [8]. Increased tissue penetration and high molar concentration may lead to increased exposure to the immune system in eyes with a potentially compromised blood retinal barrier such as neovascular AMD and diabetes [39]. The increased immunogenicity could be secondary to a variety of factors including the antibody molecule itself, the drug formulation, or due to altered post-translational protein modification. Post-translational protein modification can occur in the context of SO microdroplets (from the syringe and/or needle) as described previously, and this may be a potential exacerbating factor in the immunogenicity already observed with brolucizumab. Furthermore, the higher molar concentration of brolucizumab compared to other anti-VEGF therapies may theoretically increase the probability of these SO-protein interactions.

## Conclusions

Fundamentally, we must acknowledge eye contains immune-privileged sites with the potential for unique immune-related responses [83]. Countless mechanisms have already been put forward to explain the pathogenesis of inflammation following the injection of anti-VEGF medications. These mechanisms can be divided into 3 causes: patient-specific, medication-specific and delivery-specific. Sterile inflammation seen in association with intravitreal anti-VEGF therapy can manifest as acute onset sterile inflammation or delayed onset inflammatory vasculitis. Acute onset inflammation has a variable presentation ranging from subclinical sterile inflammation to sterile uveitis/endophthalmitis. Delayed onset inflammatory vasculitis can occur with brolucizumab and further histopathologic investigations are warranted to reach a definitive mechanism. Current knowledge of the clinical presentation and vitreous histopathology favors an auto-immune type IV hypersensitivity reaction.

The vast majority of clinically significant inflammation seen after intravitreal injection is an acute onset inflammatory response with most patients recovering baseline VA in 3–5 weeks. Physicians must be familiar with the clinical manifestations of noninfectious inflammation after an IVI in order to make a prompt differential diagnosis with endophthalmitis, choose a proper treatment, and avoid long-term complications. Further knowledge on the inflammatory vasculitis associated with brolucizumab may help guide clinicians in their clinical decision making moving forward.

This discussion underscores the role SO from syringes and needles may have on the immunogenicity of anti-VEGF after intravitreal injection. Therapeutic proteins in solution are adsorbed readily on the surface SO droplets, especially upon agitation, and this complex of protein/SO droplets as well as SO-induced protein particles can elicit immune responses [60, 61, 75]. The physician should not flick the syringe to separate air from fluid prior to injection. This contributes to SO release and this maneuver is unnecessary due to the amount of drug in the original vial [55, 56]. SO-free syringes have been studied and shown to not compromise the functional activity of ophthalmic intravitreal anti-VEGF biologics in the compounding process, and may be a safe and cost-effective alternative to siliconized syringes [84]. It is important to note that SO droplets are still possible even with SO-free syringes when a siliconized needle is attached to the syringe. There may also be inflammatory or unknown side effects of alternate lubricants used in SO-free syringes.

Although rates of intraocular inflammation related to intravitreal injection remain relatively low, the overall burden on patients remains a key concern with millions of injections performed every year [85]. Furthermore, the etiology of this ongoing issue remains an important consideration in the development of future intravitreal therapy. Our aim was to thoroughly review what is known in relation to intraocular inflammation associated with intravitreal anti-VEGF therapies to date, as knowledge regarding the causes and pathways of inflammation may lead to innovation that decreases this burden on future patients. Practical recommendations in order to minimize cases of intraocular inflammation are to avoid temperature fluctuation, mechanical shock and agitation during transport and handling of syringes and drugs, use of SO-free syringes or those with a minimal amount of SO, as all of these may contribute to the immunogenic reaction to intravitreal anti-VEGF injections.

**Table 1:** Comparison of sterile and infectious endophthalmitis

	Sterile endophthalmitis	Infectious endophthalmitis
Incidence	0.005–4.4%	0.02–0.14%
Time of onset	2.6 days (65% < 2 days)	4 days (range 0–26 days)
Presenting VA	20/150 (Range 20/25 to HM, 9% HM)	86% worse than 20/400
Severe pain	6%	74%
Hypopyon	4%	86%
Hyperemia	10%	82%
Time to resolution	3–5 weeks	Variable, depends on treatment
Prognosis	Good (15% lose > 2 lines)	Variable, usually poor

Data from references [9, 10, 17, 18, 21, 35]

**Table 2:** Characteristics of intraocular inflammation following intravitreal anti-VEGF

	Sterile uveitis/endophthalmitis	Delayed onset retinal vasculitis
Incidence	Bevacizumab (0.05–1.1%) Ranibizumab (0.005–1.9%) Aflibercept (0.05–2.1%) Brolucizumab (4.4%)^34^	Brolucizumab (0.002–3.3%)^a^
Time of onset	1–3 days	30–53 days (range 8–137)
Clinical manifestations	Decreased visual acuity Anterior chamber inflammation Vitreous cavity inflammation	Decreased visual acuity Anterior chamber inflammation Vitreous cavity inflammation Vasculitis
Final VA	Baseline VA	Mean VA loss 38 letters 46% lose > 3 lines
Attempted management	Observation Topical steroid Oral steroid Peri-ocular steroid Vitrectomy	Topical steroid Oral steroid Peri-ocular steroid Vitrectomy
Inflammatory mechanism	TASS-like reaction	Type III/IV Hypersensitivity
Potential causative factors	Drug Protein aggregates Silicone oil Endotoxin	Auto-immune reaction to drug Protein aggregates Drug impurities

Data from references [9–11, 23, 34, 35]

^a^Rate of 15.47 per 10,00 injections for retinal vasculitis and/or retinal vascular occlusion reported in cumulative review of post marketing data performed by Novartis from October 2019 through November 20th, 2020

## Data Availability

Not applicable.
